# Complete chloroplast genome of *Corylopsis spicata* and phylogenetic analysis

**DOI:** 10.1080/23802359.2019.1644223

**Published:** 2019-07-23

**Authors:** Sang-Chul Kim, Sookyung Shin, Ji-Young Ahn, Jei-Wan Lee

**Affiliations:** Department of Forest Bio Resources, National Institute of Forest Science, Suwon-si, Korea

**Keywords:** *Corylopsis spicata*, Hamamelidaceae, complete chloroplast genome, phylogenetic analysis

## Abstract

The complete plastid genome of *Corylopsis spicata* was sequenced and analyzed in this study. It was found to be 159,507 bp and consisted of a large (88,243 bp) and small (18,716 bp) single-copy regions, separated by a pair of identical inverted repeats (26,274 bp). The GC content of the whole genome was 36.9%, and there were 85 unique protein-coding genes, 37 tRNA, and eight rRNA. The gene order and organization were consistent with those of other complete plastid genomes from the Hamamelidaceae. A phylogenetic tree was constructed based on 76 protein-coding genes that demonstrated a sister relationship within the genus *Corylopsis*.

The genus *Corylopsis* Siebold & Zucc. is comprised of 26 species that are distributed in the Northern Hemisphere (Zhang and Zhang 2003). *Corylopsis spicata* Siebold & Zucc. is a species native to Japan that is mainly used for landscaping purposes because of its beautiful yellow flowers. It has very similar morphology to the Korean species *Corylopsis coreana* Uyeki but is distinguished by having yellow brown hairs in its inflorescences. In this study, we have reported for the first time, the complete sequence of the chloroplast genome of *C. spicata* and propose a molecular phylogenetic relationship in relation to the chloroplasts of other plants in the order Saxifragales.

Fresh leaves were collected from Hongneung Arboretum (37°35′N, 127°2′E) and genomic DNA was isolated from fresh leaves using a Plasmid SV mini kit (GeneAll, Seoul, Korea). Extracted DNA was stored in the Plant DNA Bank of the National Institute of Forest Science (No. 0335132684; Suwon, Korea). Whole genome sequencing was conducted using the Ion Torrent sequencing platform (Life Technologies, Carlsbad, CA). Filtered sequences were assembled using *Corylopsis coreana* as the reference sequence (GenBank accession number MG835449). The sequenced fragments were assembled using Geneious R10 (Biomatters Ltd, Auckland, New Zealand; Kearse et al. 2012). Annotation was performed using DOGMA (http://dogma.ccbb.utexas.edu/) and BLAST searches. All of the tRNA sequences were confirmed using the web-based tool tRNAScan-SE (Schattner et al. 2005) with the default settings to corroborate the tRNA boundaries identified using Geneious. The maximum likelihood (ML) tree searches and ML bootstrap searches were performed using RAxML BlackBox web-server (https://raxml-ng.vital-it.ch/#/, Stamatakis et al. 2008) with 76 protein-coding genes from 13 Saxifragales plants (outgroup: Paeoniaceae). The RAxML analyses were run with rapid bootstrap analysis using a random starting tree and 100 ML bootstrap replicates.

The plastid of *C. spicata* was found to have a double-stranded, circular DNA with 159,507 bp, and contained two inverted repeat regions (IRs) of 26,274 bp, each of which were separated by large single-copy (LSC) and small single-copy (SSC) regions of 88,243 and 18,716 bp, respectively (GenBank accession number MK942341). The genome contained 130 genes, including 85 protein-coding genes, 37 tRNA genes, and eight rRNA genes. Six of the protein-coding genes, seven tRNA genes, and four rRNA genes were duplicated in the IR regions. The overall GC content was 36.9% (LSC, 36.1%; SSC, 32.7%; IRs, 43.1%). The chloroplast of this species was very similar to that of other species of the Hamamelidaceae. The *Corylopsis* genus was found to be monophyletic (100% bootstrap values, BS) and sisters with *Loropetalum subcordatum* (Benth.) Oliv. Our results for the complete plastid genome sequence of *C. spicata* may contribute to a better understanding of the evolution of *Corylopsis* (Figure 1) and provide a useful resource for the development of species identification markers.

**Figure 1.**
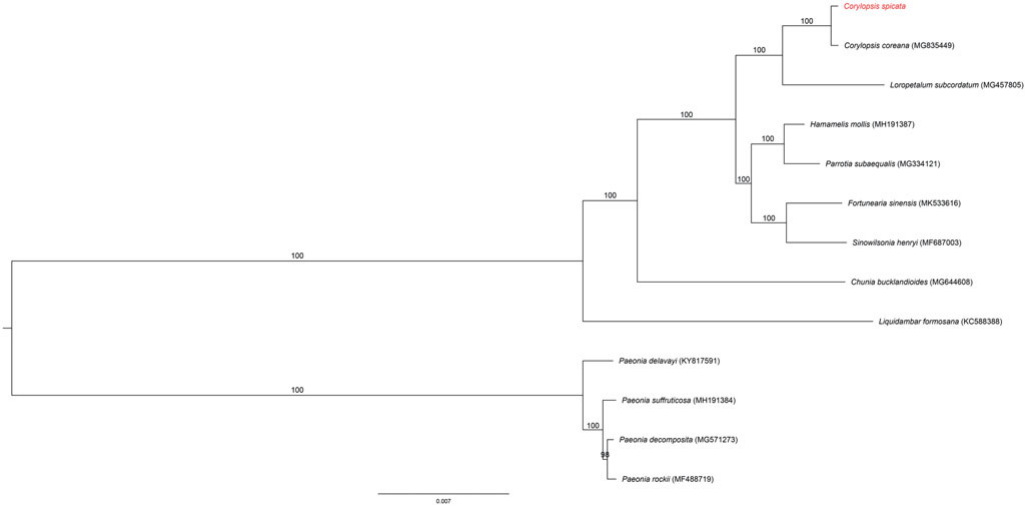
The maximum-likelihood (ML) tree based on the 13 representative chloroplast genomes of Saxifragales plants (outgroup: Paeoniaceae). The bootstrap value based on 100 replicates is shown on each node.
